# *catena*-Poly[[bis­[μ-2-(3-pyrid­yl)-1*H*-benzimidazole]-κ^2^ *N* ^2^:*N* ^3^;κ^2^ *N* ^3^:*N* ^2^-disilver(I)]-μ-2,5-dicarboxy­benzene-1,4-dicarboxyl­ato-κ^2^ *O* ^2^:*O* ^5^]

**DOI:** 10.1107/S1600536808004984

**Published:** 2008-02-27

**Authors:** Jie Chen

**Affiliations:** a Department of Civil Engineering, Fujian University of Technology, Fuzhou, Fujian 350002, People’s Republic of China

## Abstract

The title coordination polymer, [Ag_2_(C_10_H_4_O_8_)(C_12_H_9_N_3_)_2_]_*n*_, was prepared by a hydro­thermal method. The Ag^I^ atom exists in a strongly distorted trigonal coordination environment. Two Ag^I^ ions related by an inversion centre are coordinated by two 2-(3-pyrid­yl)benzimidazole ligands, forming a centrosymmetric cyclic dimer. The bridging bidentate 2,5-dicarboxy­benzene-1,4-dicarboxyl­ate ligand is also located on an inversion centre and connects the binuclear units, generating a one-dimensional polymer. The almost-planar conformation of this ligand allows it to form a strong intra­molecular O—H⋯O hydrogen bond. Finally, inter­molecular N—H⋯O hydrogen bonds aggregate the chains into a three-dimensional framework.

## Related literature

For related literature, see: Alcalde *et al.* (1992 ▶); Cao *et al.* (2002 ▶); Hu *et al.* (2004 ▶); Li *et al.* (2003 ▶); Xia *et al.* (2007 ▶).
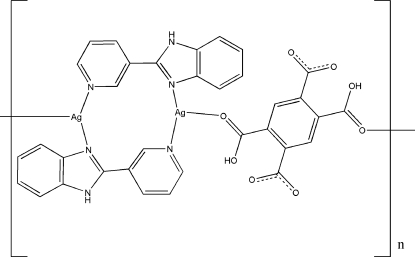


## Experimental

### 

#### Crystal data

[Ag_2_(C_10_H_4_O_8_)(C_12_H_9_N_3_)_2_]
*M* *_r_* = 858.32
Monoclinic, 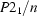

*a* = 4.8940 (11) Å
*b* = 16.011 (4) Å
*c* = 19.077 (4) Å
β = 92.393 (3)°
*V* = 1493.6 (6) Å^3^
*Z* = 2
Mo *K*α radiation
μ = 1.38 mm^−1^
*T* = 293 (2) K
0.48 × 0.13 × 0.13 mm

#### Data collection

Rigaku Mercury CCD diffractometer
Absorption correction: multi-scan (*CrystalClear*; Rigaku, 2000 ▶) *T*_min_ = 0.806, *T*_max_ = 0.842
11420 measured reflections
3405 independent reflections
3079 reflections with *I* > 2σ(*I*)
*R*_int_ = 0.023

#### Refinement

*R*[*F* ^2^ > 2σ(*F* ^2^)] = 0.030
*wR*(*F* ^2^) = 0.075
*S* = 1.07
3405 reflections
230 parameters
1 restraint
H atoms treated by a mixture of independent and constrained refinement
Δρ_max_ = 0.50 e Å^−3^
Δρ_min_ = −0.85 e Å^−3^

### 

Data collection: *CrystalClear* (Rigaku, 2000 ▶); cell refinement: *CrystalClear*; data reduction: *CrystalClear*; program(s) used to solve structure: *SHELXS97* (Sheldrick, 2008 ▶); program(s) used to refine structure: *SHELXL97* (Sheldrick, 2008 ▶); molecular graphics: *CrystalStructure* (Rigaku, 2000 ▶); software used to prepare material for publication: *SHELXL97*.

## Supplementary Material

Crystal structure: contains datablocks I, global. DOI: 10.1107/S1600536808004984/bh2158sup1.cif

Structure factors: contains datablocks I. DOI: 10.1107/S1600536808004984/bh2158Isup2.hkl

Additional supplementary materials:  crystallographic information; 3D view; checkCIF report

## Figures and Tables

**Table:** 

Ag1—N2	2.1663 (18)
Ag1—N1^i^	2.207 (2)
Ag1—O1	2.3889 (18)

**Table:** 

N2—Ag1—N1^i^	150.61 (7)
N2—Ag1—O1	115.06 (7)
N1^i^—Ag1—O1	93.66 (7)

Symmetry code: (i) 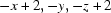.

**Table 2:** Hydrogen-bond geometry (Å, °)

*D*—H⋯*A*	*D*—H	H⋯*A*	*D*⋯*A*	*D*—H⋯*A*
O2—H2*B*⋯O4	0.846 (19)	1.56 (2)	2.399 (3)	171 (4)
N3—H3*A*⋯O3^ii^	0.86	1.92	2.742 (3)	159

Symmetry code: (ii) 
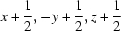
.

**Fig. 1.**
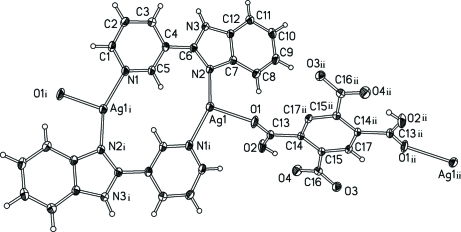
The structure of (I), with the atomic labels and 30% probability displacement ellipsoids for non-H atoms. [Symmetry codes: (i) 2 - x, -y, 2 - z; (ii) 1 - x, 1 - y, 2 - z].

**Fig. 2.**
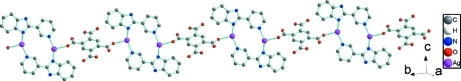
A view of the one-dimensional chain of (I). H atoms are omitted for clarity.

**Fig. 3.**
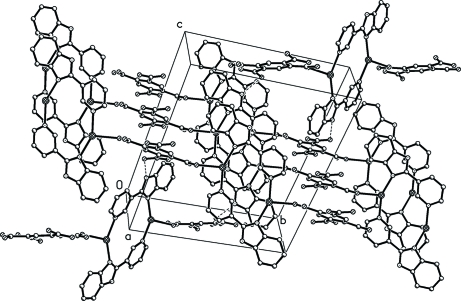
The crystal packing of (I). H atoms are omitted for clarity.

## supplementary crystallographic information

### Comment

1,2,4,5-Benzenetetracarboxylic acid (H_4_BTEC) has been regarded as an excellent candidate for the construction of multi-dimensional coordination polymers for its versatile coordination modes and rich hydrogen bonding (Cao *et al.*, 2002; Hu *et al.*, 2004; Li *et al.*, 2003). The coordination supramolecular architectures are controlled by the extent of the deprotonation of H_4_BTEC, nature of the auxiliary ligands, and metal coordination centers.

In the title coordination polymer, (I), the Ag^I^ is three coordinated (Fig. 1). Two symmetry related Ag^I^ ions are bonded by four N atoms from two 2-(3-pyridyl)-1*H*-benzimidazole ligands in the head to end mode, forming a centrosymmetric cyclic dimer. The coordination sphere of the cyclic dimer is completed by the 2,5-dicarboxybenzene-1,4-dicarboxylate coordinating in a bis(monodentate) fashion, which is similar to the coordination mode reported by Xia *et al.* (2007). The centrosymmetric bridging 2,5-dicarboxybenzene-1,4-dicarboxylate ligands link the binuclear units into a one-dimensional polymeric chain (Fig. 2). Interestingly, the coordinating O atom is provided by the undeprotonated carboxylic group rather than the deprotonated one. The O2—H group in the undeprotonated carboxylic group forms a strong intramolecular hydrogen bond with the adjacent carboxylate group [O2···O4: 2.399 (3) Å]. This contact allows planarity for the bridging ligand, which may be related to the non-coordinating character of carboxylate functionalities. The uncoordinated carboxylate group also provides another O atom for the formation of intermolecular hydrogen bonds [N3···O3: 2.742 (3) Å], forming a three-dimensional framework (Fig. 3). Despite of the presence of aromatic rings, no apparent π···π stacking interactions are found in the crystal structure.

### Experimental

A solution of Ag_2_O (0.07 g, 0.30 mmol), 2-(3-pyridyl)-1*H*-benzimidazole (Alcalde *et al.*, 1992) (0.14 g, 0.61 mmol), 1,2,4,5-benzenetetracarboxylic acid (0.066 g, 0.30 mmol) and H_2_O (15 ml) was stirred under ambient conditions. The solution was sealed in a 25 ml Teflon-lined stainless steel vessel, heated at 413 K for 4 days and cooled to room temperature for 3 days. The resulting product was recovered by filtration, washed with distilled water and dried in air (65% yield).

### Refinement

Anisotropic thermal parameters were applied to all non-hydrogen atoms. The carboxylic acid H atom H2B was initially located in a difference map, and then refined with a restrained O—H bond length of 0.83 (1) Å. Other H atoms were fixed geometrically and allowed to ride on their parent atoms, with C—H = 0.93 Å, N—H = 0.86 Å, and *U*_iso_(H) = 1.2*U*_eq_(carrier atom).

### Crystal data

**Table:**

[Ag_2_(C_10_H_4_O_8_)(C_12_H_9_N_3_)_2_]	*F*_000_ = 852
*M**_r_* = 858.32	*D*_x_ = 1.909 Mg m^−^^3^
Monoclinic, *P*2_1_/*n*	Mo *K*α radiation λ = 0.71073 Å
Hall symbol: -P 2yn	Cell parameters from 3641 reflections
*a* = 4.8940 (11) Å	θ = 2.5–27.5º
*b* = 16.011 (4) Å	µ = 1.38 mm^−^^1^
*c* = 19.077 (4) Å	*T* = 293 (2) K
β = 92.393 (3)º	Prism, colourless
*V* = 1493.6 (6) Å^3^	0.48 × 0.13 × 0.13 mm
*Z* = 2	

### Data collection

**Table:**

Rigaku Mercury CCD diffractometer	3405 independent reflections
Radiation source: fine-focus sealed tube	3079 reflections with *I* > 2σ(*I*)
Monochromator: graphite	*R*_int_ = 0.023
*T* = 293(2) K	θ_max_ = 27.5º
ω scans	θ_min_ = 2.5º
Absorption correction: multi-scan(CrystalClear; Rigaku, 2000)	*h* = −6→6
*T*_min_ = 0.806, *T*_max_ = 0.842	*k* = −20→20
11420 measured reflections	*l* = −18→24

### Refinement

**Table:**

Refinement on *F*^2^	Secondary atom site location: difference Fourier map
Least-squares matrix: full	Hydrogen site location: inferred from neighbouring sites
*R*[*F*^2^ > 2σ(*F*^2^)] = 0.030	H atoms treated by a mixture of independent and constrained refinement
*wR*(*F*^2^) = 0.075	*w* = 1/[σ^2^(*F*_o_^2^) + (0.0366*P*)^2^ + 0.9191*P*] where *P* = (*F*_o_^2^ + 2*F*_c_^2^)/3
*S* = 1.07	(Δ/σ)_max_ = 0.005
3405 reflections	Δρ_max_ = 0.50 e Å^−^^3^
230 parameters	Δρ_min_ = −0.85 e Å^−^^3^
1 restraint	Extinction correction: none
Primary atom site location: structure-invariant direct methods	

### Fractional atomic coordinates and isotropic or equivalent isotropic displacement parameters (Å^2^)

**Table:**

	*x*	*y*	*z*	*U*_iso_*/*U*_eq_	
Ag1	0.71244 (4)	0.134099 (11)	1.018347 (10)	0.04125 (9)	
C1	1.1680 (5)	−0.15500 (14)	1.13812 (13)	0.0343 (5)	
H1A	1.2997	−0.1963	1.1336	0.041*	
C2	1.0649 (5)	−0.14053 (14)	1.20320 (14)	0.0359 (5)	
H2A	1.1275	−0.1717	1.2418	0.043*	
C3	0.8688 (5)	−0.07976 (15)	1.21087 (12)	0.0329 (5)	
H3B	0.7980	−0.0695	1.2545	0.039*	
C4	0.7781 (4)	−0.03380 (13)	1.15231 (12)	0.0265 (4)	
C5	0.8913 (5)	−0.05275 (13)	1.08884 (12)	0.0324 (5)	
H5A	0.8300	−0.0232	1.0493	0.039*	
C6	0.5728 (4)	0.03242 (13)	1.15806 (11)	0.0264 (4)	
C7	0.3021 (5)	0.13812 (13)	1.13776 (12)	0.0269 (4)	
C8	0.1575 (5)	0.20687 (14)	1.11051 (13)	0.0347 (5)	
H8A	0.1860	0.2269	1.0656	0.042*	
C9	−0.0299 (5)	0.24392 (16)	1.15313 (14)	0.0386 (6)	
H9A	−0.1287	0.2900	1.1365	0.046*	
C10	−0.0745 (5)	0.21380 (16)	1.22058 (14)	0.0383 (6)	
H10A	−0.2039	0.2399	1.2474	0.046*	
C11	0.0682 (5)	0.14644 (15)	1.24842 (13)	0.0332 (5)	
H11A	0.0396	0.1267	1.2934	0.040*	
C12	0.2577 (4)	0.10963 (13)	1.20541 (11)	0.0264 (4)	
C13	0.5075 (5)	0.32005 (13)	0.96973 (12)	0.0322 (5)	
C14	0.4884 (5)	0.41435 (13)	0.98108 (11)	0.0271 (4)	
C15	0.3175 (5)	0.47238 (13)	0.94531 (11)	0.0271 (4)	
C16	0.1144 (5)	0.45603 (15)	0.88456 (12)	0.0320 (5)	
C17	0.3352 (5)	0.55568 (14)	0.96571 (12)	0.0297 (5)	
H17A	0.2214	0.5939	0.9422	0.036*	
N1	1.0845 (4)	−0.11132 (12)	1.08101 (11)	0.0347 (4)	
N2	0.5015 (4)	0.08847 (11)	1.10831 (9)	0.0273 (4)	
N3	0.4299 (4)	0.04296 (11)	1.21627 (9)	0.0273 (4)	
H3A	0.4443	0.0131	1.2537	0.033*	
O1	0.6918 (4)	0.28180 (11)	1.00119 (11)	0.0484 (5)	
O2	0.3341 (4)	0.28260 (11)	0.92949 (12)	0.0486 (5)	
H2B	0.217 (7)	0.316 (2)	0.912 (2)	0.082 (13)*	
O3	0.0298 (4)	0.51666 (11)	0.84962 (9)	0.0440 (4)	
O4	0.0356 (4)	0.38153 (11)	0.87095 (10)	0.0456 (5)	

### Atomic displacement parameters (Å^2^)

**Table:**

	*U*^11^	*U*^22^	*U*^33^	*U*^12^	*U*^13^	*U*^23^
Ag1	0.05515 (15)	0.02899 (12)	0.04086 (13)	0.01185 (8)	0.01680 (9)	0.00789 (7)
C1	0.0358 (12)	0.0225 (10)	0.0449 (14)	0.0059 (9)	0.0042 (10)	0.0043 (9)
C2	0.0383 (13)	0.0301 (12)	0.0393 (13)	0.0058 (10)	0.0011 (10)	0.0093 (9)
C3	0.0362 (12)	0.0305 (12)	0.0320 (12)	0.0041 (10)	0.0018 (9)	0.0010 (9)
C4	0.0274 (11)	0.0187 (9)	0.0333 (11)	−0.0010 (8)	0.0010 (8)	−0.0001 (8)
C5	0.0411 (13)	0.0216 (10)	0.0347 (12)	0.0054 (9)	0.0037 (10)	0.0026 (8)
C6	0.0283 (11)	0.0205 (10)	0.0303 (11)	−0.0005 (8)	−0.0007 (8)	−0.0007 (8)
C7	0.0263 (11)	0.0219 (10)	0.0323 (11)	−0.0002 (8)	−0.0002 (8)	−0.0008 (8)
C8	0.0368 (13)	0.0280 (11)	0.0386 (13)	0.0034 (10)	−0.0037 (10)	0.0050 (9)
C9	0.0372 (13)	0.0284 (12)	0.0492 (15)	0.0097 (10)	−0.0073 (11)	−0.0006 (10)
C10	0.0324 (12)	0.0360 (13)	0.0463 (14)	0.0081 (10)	0.0001 (10)	−0.0107 (10)
C11	0.0319 (12)	0.0337 (12)	0.0339 (12)	0.0015 (9)	0.0014 (9)	−0.0027 (9)
C12	0.0259 (11)	0.0223 (10)	0.0307 (11)	0.0005 (8)	−0.0023 (8)	−0.0012 (8)
C13	0.0455 (14)	0.0189 (10)	0.0327 (12)	0.0017 (9)	0.0057 (10)	−0.0001 (8)
C14	0.0379 (12)	0.0173 (9)	0.0261 (10)	0.0030 (8)	0.0037 (9)	0.0002 (7)
C15	0.0347 (12)	0.0219 (10)	0.0249 (10)	0.0022 (9)	0.0035 (8)	0.0003 (8)
C16	0.0380 (13)	0.0309 (11)	0.0269 (11)	0.0029 (9)	0.0012 (9)	−0.0016 (9)
C17	0.0400 (13)	0.0207 (10)	0.0282 (11)	0.0055 (9)	−0.0013 (9)	0.0031 (8)
N1	0.0428 (12)	0.0208 (9)	0.0410 (11)	0.0040 (8)	0.0082 (9)	0.0028 (8)
N2	0.0303 (10)	0.0220 (9)	0.0297 (9)	0.0025 (7)	0.0020 (7)	0.0016 (7)
N3	0.0304 (10)	0.0244 (9)	0.0272 (9)	0.0038 (7)	0.0014 (7)	0.0023 (7)
O1	0.0691 (13)	0.0194 (8)	0.0554 (11)	0.0126 (8)	−0.0126 (10)	0.0003 (7)
O2	0.0545 (12)	0.0220 (8)	0.0680 (13)	0.0009 (8)	−0.0133 (10)	−0.0091 (8)
O3	0.0638 (12)	0.0335 (9)	0.0334 (9)	0.0062 (9)	−0.0137 (8)	−0.0009 (7)
O4	0.0538 (12)	0.0345 (9)	0.0471 (11)	−0.0065 (8)	−0.0147 (9)	−0.0035 (8)

### Geometric parameters (Å, °)

**Table:**

Ag1—N2	2.1663 (18)	C9—H9A	0.9300
Ag1—N1^i^	2.207 (2)	C10—C11	1.379 (3)
Ag1—O1	2.3889 (18)	C10—H10A	0.9300
C1—N1	1.344 (3)	C11—C12	1.394 (3)
C1—C2	1.379 (4)	C11—H11A	0.9300
C1—H1A	0.9300	C12—N3	1.370 (3)
C2—C3	1.379 (3)	C13—O1	1.226 (3)
C2—H2A	0.9300	C13—O2	1.271 (3)
C3—C4	1.395 (3)	C13—C14	1.529 (3)
C3—H3B	0.9300	C14—C17^ii^	1.390 (3)
C4—C5	1.386 (3)	C14—C15	1.407 (3)
C4—C6	1.468 (3)	C15—C17	1.391 (3)
C5—N1	1.344 (3)	C15—C16	1.518 (3)
C5—H5A	0.9300	C16—O3	1.239 (3)
C6—N3	1.347 (3)	C16—O4	1.277 (3)
C6—N2	1.342 (3)	C16—O4	1.277 (3)
C7—C8	1.397 (3)	C17—H17A	0.9300
C7—N2	1.395 (3)	N3—O3^iii^	2.742 (3)
C7—C12	1.394 (3)	N3—H3A	0.8600
C8—C9	1.384 (4)	O2—O4	2.399 (3)
C8—H8A	0.9300	O2—H2B	0.846 (19)
C9—C10	1.400 (4)		
			
N2—Ag1—N1^i^	150.61 (7)	C12—C11—H11A	121.9
N2—Ag1—O1	115.06 (7)	N3—C12—C11	131.3 (2)
N1^i^—Ag1—O1	93.66 (7)	N3—C12—C7	106.02 (18)
N1—C1—C2	122.1 (2)	C11—C12—C7	122.7 (2)
N1—C1—H1A	118.9	O1—C13—O2	121.3 (2)
C2—C1—H1A	118.9	O1—C13—C14	118.2 (2)
C3—C2—C1	119.8 (2)	O2—C13—C14	120.5 (2)
C3—C2—H2A	120.1	C17^ii^—C14—C15	117.68 (19)
C1—C2—H2A	120.1	C17^ii^—C14—C13	113.86 (19)
C2—C3—C4	119.1 (2)	C15—C14—C13	128.5 (2)
C2—C3—H3B	120.4	C17—C15—C14	117.9 (2)
C4—C3—H3B	120.4	C17—C15—C16	114.22 (19)
C5—C4—C3	117.3 (2)	C14—C15—C16	127.83 (19)
C5—C4—C6	121.6 (2)	O3—C16—O4	122.1 (2)
C3—C4—C6	121.1 (2)	O3—C16—O4	122.1 (2)
N1—C5—C4	124.0 (2)	O3—C16—C15	118.0 (2)
N1—C5—H5A	118.0	O4—C16—C15	119.9 (2)
C4—C5—H5A	118.0	O4—C16—C15	119.9 (2)
N3—C6—N2	111.75 (19)	C15—C17—C14^ii^	124.4 (2)
N3—C6—C4	122.18 (19)	C15—C17—H17A	117.8
N2—C6—C4	126.1 (2)	C14^ii^—C17—H17A	117.8
C8—C7—N2	130.6 (2)	C5—N1—C1	117.7 (2)
C8—C7—C12	120.4 (2)	C5—N1—Ag1^i^	123.69 (16)
N2—C7—C12	108.97 (19)	C1—N1—Ag1^i^	118.53 (16)
C9—C8—C7	117.2 (2)	C6—N2—C7	105.18 (18)
C9—C8—H8A	121.4	C6—N2—Ag1	131.88 (15)
C7—C8—H8A	121.4	C7—N2—Ag1	119.44 (14)
C8—C9—C10	121.7 (2)	C6—N3—C12	108.07 (18)
C8—C9—H9A	119.2	C6—N3—O3^iii^	129.98 (14)
C10—C9—H9A	119.2	C12—N3—O3^iii^	119.74 (14)
C11—C10—C9	121.9 (2)	C6—N3—H3A	126.0
C11—C10—H10A	119.1	C12—N3—H3A	126.0
C9—C10—H10A	119.1	C13—O1—Ag1	125.98 (17)
C10—C11—C12	116.3 (2)	C13—O2—O4	110.37 (15)
C10—C11—H11A	121.9	C13—O2—H2B	111 (3)
			
N1—C1—C2—C3	0.3 (4)	C14—C15—C17—C14^ii^	0.3 (4)
C1—C2—C3—C4	0.0 (4)	C16—C15—C17—C14^ii^	−178.4 (2)
C2—C3—C4—C5	0.4 (3)	C4—C5—N1—C1	1.3 (4)
C2—C3—C4—C6	−179.0 (2)	C4—C5—N1—Ag1^i^	−174.85 (17)
C3—C4—C5—N1	−1.1 (4)	C2—C1—N1—C5	−0.9 (4)
C6—C4—C5—N1	178.3 (2)	C2—C1—N1—Ag1^i^	175.50 (19)
C5—C4—C6—N3	169.4 (2)	N3—C6—N2—C7	0.7 (2)
C3—C4—C6—N3	−11.2 (3)	C4—C6—N2—C7	−179.8 (2)
C5—C4—C6—N2	−10.2 (3)	N3—C6—N2—Ag1	158.67 (15)
C3—C4—C6—N2	169.2 (2)	C4—C6—N2—Ag1	−21.7 (3)
N2—C7—C8—C9	−179.8 (2)	C8—C7—N2—C6	179.0 (2)
C12—C7—C8—C9	−0.6 (3)	C12—C7—N2—C6	−0.3 (2)
C7—C8—C9—C10	−0.3 (4)	C8—C7—N2—Ag1	17.7 (3)
C8—C9—C10—C11	0.9 (4)	C12—C7—N2—Ag1	−161.64 (14)
C9—C10—C11—C12	−0.5 (4)	N1^i^—Ag1—N2—C6	57.2 (3)
C10—C11—C12—N3	−179.8 (2)	O1—Ag1—N2—C6	−135.92 (19)
C10—C11—C12—C7	−0.4 (4)	N1^i^—Ag1—N2—C7	−147.28 (17)
C8—C7—C12—N3	−179.5 (2)	O1—Ag1—N2—C7	19.57 (18)
N2—C7—C12—N3	−0.1 (2)	N2—C6—N3—C12	−0.8 (2)
C8—C7—C12—C11	0.9 (4)	C4—C6—N3—C12	179.6 (2)
N2—C7—C12—C11	−179.7 (2)	N2—C6—N3—O3^iii^	−163.40 (15)
O1—C13—C14—C17^ii^	−8.4 (3)	C4—C6—N3—O3^iii^	17.0 (3)
O2—C13—C14—C17^ii^	170.6 (2)	C11—C12—N3—C6	−180.0 (2)
O1—C13—C14—C15	172.0 (2)	C7—C12—N3—C6	0.5 (2)
O2—C13—C14—C15	−9.0 (4)	C11—C12—N3—O3^iii^	−15.2 (3)
C17^ii^—C14—C15—C17	−0.3 (4)	C7—C12—N3—O3^iii^	165.26 (14)
C13—C14—C15—C17	179.3 (2)	O2—C13—O1—Ag1	−15.7 (4)
C17^ii^—C14—C15—C16	178.2 (2)	C14—C13—O1—Ag1	163.34 (15)
C13—C14—C15—C16	−2.1 (4)	N2—Ag1—O1—C13	−85.5 (2)
C17—C15—C16—O3	15.1 (3)	N1^i^—Ag1—O1—C13	88.1 (2)
C14—C15—C16—O3	−163.5 (2)	O1—C13—O2—O4	−175.0 (2)
C17—C15—C16—O4	−164.8 (2)	C14—C13—O2—O4	6.0 (3)
C14—C15—C16—O4	16.6 (4)	O3—C16—O4—O4	0.0 (4)
C17—C15—C16—O4	−164.8 (2)	C15—C16—O4—O4	0.0 (3)
C14—C15—C16—O4	16.6 (4)		

Symmetry codes: (i) −*x*+2, −*y*, −*z*+2; (ii) −*x*+1, −*y*+1, −*z*+2;  (iii) *x*+1/2, −*y*+1/2, *z*+1/2.

### Hydrogen-bond geometry (Å, °)

**Table:**

*D*—H···*A*	*D*—H	H···*A*	*D*···*A*	*D*—H···*A*
O2—H2B···O4	0.846 (19)	1.56 (2)	2.399 (3)	171 (4)
N3—H3A···O3^iii^	0.86	1.92	2.742 (3)	159

Symmetry codes:  (iii) *x*+1/2, −*y*+1/2, *z*+1/2.

## References

[bb1] Alcalde, E., Dinarés, I., Pérez-García, L. & Roca, T. (1992). *Synthesis*, pp. 395–398.

[bb2] Cao, R., Sun, D., Liang, Y., Hong, M., Tatsumi, K. & Shi, Q. (2002). *Inorg. Chem.* **41**, 2087–2094. 10.1021/ic0110124 11952362

[bb3] Hu, M.-L., Xiao, H.-P. & Yuan, J.-X. (2004). *Acta Cryst.* C**60**, m112–m113. 10.1107/S0108270103029445 15004359

[bb4] Li, Y., Hao, N., Lu, Y., Wang, E., Kang, Z. & Hu, C. (2003). *Inorg. Chem.* **42**, 3119–3124. 10.1021/ic026306j 12716210

[bb5] Rigaku (2000). *CrystalStructure* (Version 3.7.0) and *CrystalClear* (Version 1.36). Rigaku Corporation, Tokyo, Japan.

[bb6] Sheldrick, G. M. (2008). *Acta Cryst.* A**64**, 112–122. 10.1107/S0108767307043930 18156677

[bb7] Xia, C.-K., Wu, W., Qiu, L. & Xie, J.-M. (2007). *Acta Cryst.* E**63**, m2881.

